# Evaluation and management of nonarteritic anterior ischemic optic neuropathy: a national survey

**DOI:** 10.1007/s00417-024-06512-y

**Published:** 2024-05-15

**Authors:** Omer Y. Bialer, Hadas Stiebel-Kalish

**Affiliations:** 1 Ophthalmology Department, Rabin Medical Center, 39th Jabotinsky Street, Petah-Tikva, Israel; 2 School of Medicine, Tel-Aviv University, Tel-Aviv, Israel

**Keywords:** Nonarteritic anterior ischemic optic neuropathy (NAION), Neuro-ophthalmologists, Expert opinion, Optic neuropathy

## Abstract

**Purpose:**

The evaluation and management of Nonarteritic Anterior Ischemic Optic Neuropathy (NAION) lacks standardized guidelines. This study aimed to investigate the real-world practices of neuro-ophthalmologists in the evaluation and management of typical NAION cases.

**Methods:**

A national survey, conducted between 2019 and 2021, involved all practicing neuro-ophthalmologists. A structured questionnaire assessed their approach to risk factor evaluation and treatment of NAION, with 19 questions about risk factors and six questions concerning treatment and prevention of fellow-eye involvement.

**Results:**

Thirty-six out of 37 neuro-ophthalmologists participated. Most physicians referred patients for evaluation of the following risk factors: obstructive sleep apnea (83.3%), diabetes mellitus (83.3%), hypertension (77.7%), dyslipidemia (72.2%), and optic disc drusen (38.8%). However, there was considerable variation in the choice of diagnostic tests recommended. Furthermore, nearly 47% recommended an embolism workup. Regarding treatment, the majority (91%) did not recommend routine treatment for NAION, although in 16.7%, high-dose corticosteroids were occasionally prescribed. Secondary prevention with aspirin (80.6%), smoking cessation advice (86.1%), and advising against erectile dysfunction medications for men (80.6%) were common recommendations.

**Conclusion:**

While the risk factors associated with NAION are well-reported, there is a lack of uniformity on which tests should be ordered to evaluate these risk factors. Most neuro-ophthalmologists concur that routine treatment for NAION is not warranted, but not unanimously. Future studies to develop a consensus guideline for post-NAION work-up and management recommendations may assist in the detection and management of preventable risk factors.
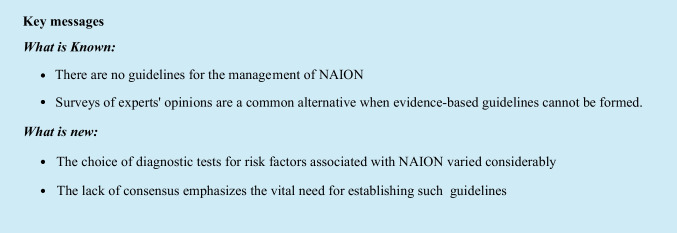

**Supplementary Information:**

The online version contains supplementary material available at 10.1007/s00417-024-06512-y.

## Introduction

Nonarteritic anterior ischemic optic neuropathy (NAION) is the most prevalent acute optic neuropathy affecting middle-aged and older individuals [1, 2]. Typical NAION manifests as a sudden, mostly painless monocular visual loss, often noticed upon awakening, with progression occurring over a span of days or weeks [1, 3]. Despite being a common cause of visual impairment [4], no current treatment exists [5, 6]. Management is aimed at decreasing the risk for fellow-eye NAION and improving overall cardiovascular health through control of associated risk factors common for both disorders.

The pathophysiology of NAION remains elusive. The leading theory implicates hypoperfusion of branches of the short posterior ciliary arteries that supply the optic nerve head. This sets in motion a vicious cycle involving axoplasmic flow stasis and axonal swelling, subsequently compressing small vessels within the confined space of a small optic disc (often referred to as the "disc at risk"), leading to aggravated ischemia [1, 7].

Debate persists concerning the risk factors associated with idiopathic NAION [8–12]. Commonly accepted risk factors include age and a crowded optic disc, along with conditions like diabetes mellitus (DM), hypertension (HTN), dyslipidemia, obstructive sleep apnea (OSA), obesity, nocturnal hypotension, smoking, optic disc drusen (ODD), and specific medications including erectile dysfunction medications, amiodarone, and interferon [13]. No consensus exists to guide neuro-ophthalmologists on the best means to diagnose these commonly accepted risk factors for NAION.

Surveys examining routine management are a common alternative when evidence-based guidelines cannot be formed [14–18]. Although these surveys lack objective quantitative outcomes, they offer valuable insights based on the collective experience of physicians within the field.

This study addresses a fundamental question: What constitutes the customary (routine) neuro-ophthalmological work-up and management of NAION? This query holds particular relevance given the absence of established guidelines and international consensus among neuro-ophthalmologists regarding the standard evaluation and management of NAION [13, 19]. As such, a pragmatic approach may involve adhering to the prevailing practices.

## Methods

### Study participants and data collection

This was a national survey of neuro-ophthalmologists in Israel. All fellowship-trained neuro-ophthalmologists actively practicing between 2019 and 2021 in Israel were contacted and invited to participate in the study. Their professional attributes are presented in Supplemental Table 1. Those who consented were contacted by phone. They were provided with a clinical scenario depicting a patient with a typical presentation of NAION, which included the following characteristics:

1. An adult patient with acute, painless monocular visual loss (age and sex were not specified).
2. Unilateral optic neuropathy characterized by disc edema and peripapillary hemorrhages, with a crowded optic disc ("disc at risk") noted in the fellow eye.
3. Absence of arteritic symptoms and normal Erythrocyte Sedimentation Rate (ESR) and C-reactive protein (CRP) levels.
4. No history of head trauma or signs of orbital pathology.

### Questionnaire design and administration

Participants were then presented with a structured questionnaire consisting of 19 questions related to the investigation of risk factors for NAION and six questions concerning treatment and patient instructions. The questionnaire is presented as supplemental Table 2. For each diagnostic or therapeutic step, participants were requested to respond "yes," "no," or "depends," on whether they recommend the procedure as part of *routine* practice for *all or most* of their patients with NAION. For instances they responded “depends”, participants were asked to specify the circumstances under which they would recommend the step. Participants were also encouraged to describe any diagnostic or therapeutic measures not covered in the questionnaire.

Using a questionnaire in this format was chosen to align with the primary purpose of this study: to identify commonalities and differences among neuro-ophthalmologists in evaluating typical NAION patients. This questionnaire avoids influencing or criticizing the neuro-ophthalmologists' responses while enabling the grouping of individual responses into easily understandable categories. The questionnaire utilized in this study has not been previously validated. However, it does not produce a numerical or categorical outcome (e.g. a total score or risk assessment) but was designed solely to facilitate uniformity in the collection of data during the telephone interviews (i.e. ensuring that all participants were consistently asked the same questions).

### Data collection and analysis

Recorded responses from the participating neuro-ophthalmologists were stored in an Excel spreadsheet. The analysis included the identification of common trends, preferences, and variations in clinical practice among the surveyed experts.

## Results

**Fig. 1:**
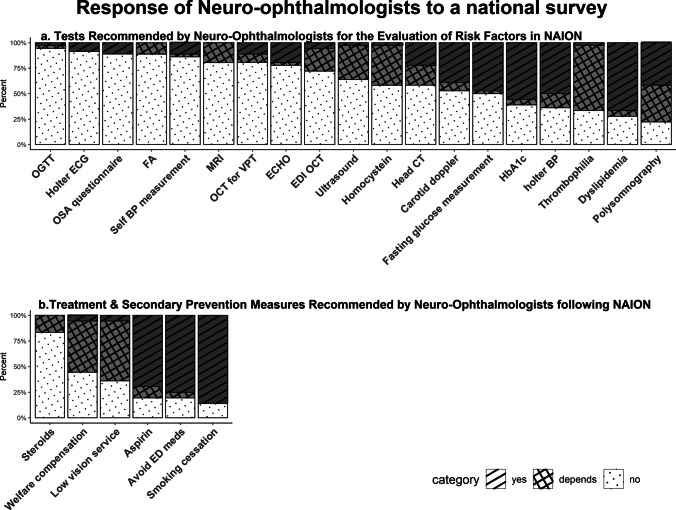
Participants were asked if they performed each test or treatment to all their patients with typical NAION and were required to answer "yes", "no" or "depends" (i.e. sometimes). Responses are presented as percentages out of 36 participants, in descending order according to the frequency of responding "no". The figure was created with R: a language and environment for statistical computing (R Foundation for Statistical Computing, http://www.R-project.org) and GIMP software (version 2.10.32), an open-source image manipulation program. Fig. 1A presents the specific diagnostic tests neuro-ophthalmologists perform in the evaluation of patients with NAION. Figure. 1B presents the specific treatments and lifestyle recommendations of the participating neuro-ophthalmologists. OGTT= Oral Glucose Tolerance Test. OSA= Obstructive Sleep Apnea. FA= Fluorescein Angiography. BP= Blood Pressure. VPT= Vitreo-Papillary Traction. EDI= Enhanced Depth Imaging. ED meds= Erectile Dysfunction medications

Between the years 2019 and 2021, there were 37 active fellowship-trained neuro-ophthalmologists in Israel. Thirty-six (97.3%) of these specialists consented to participate in our study. Figure 1 presents the accumulated responses of participants. The evaluation of risk factors is graphically summarized in Fig. 1A.

### Cardiovascular risk factors

Three out of 36 (8.3%) referred patients to their general practitioner for diagnosis and evaluation of all cardiovascular conditions without a specific recommendation.

#### Obstructive Sleep Apnea

Thirty out of 36 (83.3%), evaluated patients for OSA. Among them, four (11.1%) used the Berlin questionnaire for OSA screening, while 15 (41.6%) relied on unstructured questions about OSA symptoms (e.g., snoring, morning fatigue, history of apnea). Furthermore, 15 (41.6%) referred all their patients for polysomnography, and 13 (36.1%) selectively referred patients exhibiting OSA symptoms or characteristic physical traits (e.g., high body mass index and neck circumference) [20, 21].

#### Diabetes Mellitus

Thirty of 36 (83.3%) neuro-ophthalmologists conducted DM assessments. Of these, 18 (50%) consistently obtained fasting glucose levels, with one doing so exclusively for patients without a prior DM diagnosis. Additionally, two (5.6%) referred patients without prior DM diagnosis for a glucose tolerance test. Twenty-two (61.1%) routinely acquired serum HbA1c levels, with two (5.6%) doing so only for patients without a medical history of DM. Among them, 16 (53.3%) recommended either fasting glucose or HbA1c testing exclusively.

#### Hypertension

Twenty-eight (77.7%) neuro-ophthalmologists routinely assessed patients for HTN. Notably, five (13.8%) recommended self-blood pressure (BP) monitoring at home, with one suggesting it only when the patient appeared capable of performing it. Eighteen (50%), referred all patients for a 24-h Holter BP monitoring. An additional five (13.9%) recommended this monitoring only under specific conditions, such as when HTN was previously unknown or in younger patients.

#### Dyslipidemia

Twenty-six out of 36 physicians (72.2%) obtained fasting serum lipid profiles. Two of them reserved this test for patients without a previous diagnosis of dyslipidemia.

Less frequently recommended tests included a 24-h Holter ECG (3, 8.3%), carotid Doppler ultrasound (17, 47.2%), and echocardiogram (8, 22.2%). Most of them obtained these tests routinely (for each test, one physician recommended the test only under specific circumstances such as when retinal arterial narrowing was observed on funduscopy or when no other risk factors were evident).

Additionally, blood tests for thrombophilia were suggested by 24 out of 36 (66.6%) neuro-ophthalmologists, with only one performing this routinely. Most of them considered young age (< 45) as an indication for thrombophilia screening. Three conducted it in cases with atypical presentations, such as bilateral simultaneous NAION, and one did it only when no cardiovascular risk factors were apparent. Serum homocysteine levels were specifically assessed by 15 (41.6%) neuro-ophthalmologists, although only one recommended this test routinely.

### Ocular structural risk factors

Fourteen (38.8%) physicians regularly ruled out optic disc drusen. Among them, five (13.8%) referred patients for ocular sonography, one (2.7%) referred them exclusively for EDI-OCT of the optic disc, and eight (22.2%) obtained both ultrasound and EDI-OCT. Vitreo-papillary traction was specifically evaluated in an OCT of the optic nerve head by seven (19.4%) neuro-ophthalmologists, although only four (11.1%) referred their patients for this imaging on a routine basis.

### Imaging

Head computed tomography (CT) with contrast was ordered for all patients with NAION by eight (22.2%) neuro-ophthalmologists. Seven (19.4%) referred patients to a head CT only for specific indications; five for patients younger than 45 years old and two for atypical clinical presentations. Seven neuro-ophthalmologists (19.4%) referred patients for an MRI of the head and orbits with gadolinium, but again, only in specific indications; three for patients younger than 45, two for patients with a history of malignancy, one for atypical clinical presentations, and one obtained an MRI when no risk factors were known. Only two neuro-ophthalmologists considered obtaining both a CT and MRI, while 20 opted for one or the other. Fluorescein angiography in the acute stage was not a routine practice, with only four (11.1%) performing it infrequently. No other ancillary tests were consistently recommended by more than one neuro-ophthalmologist.

### Treatment and fellow eye risk-reduction

Responses are detailed in Fig. 1B. Low-dose aspirin was recommended by 29 of 36 (80.5%) neuro-ophthalmologists. Of these, 25 (69.4%) recommended it for all patients not previously on anti-platelet medications, while four (11.1%) suggested it only in the presence of cardiovascular risk factors or with the support of the general physician.

Steroids were not routinely prescribed by any neuro-ophthalmologist for patients with NAION. However, six (16.7%) offered their patients intravenous steroids in cases of second-eye involvement, particularly when severe vision loss occurred in the first eye. Additional indications included progressive NAION (one respondent) or young age (one respondent).

Additional treatments were reported by only a few practitioners, with one recommending intravitreal bevacizumab for patients with severe vision loss within seven days of symptom onset. Two (5.5%) prescribed topical anti-glaucoma drops, one utilizing brinzolamide for a month and the other employing brimonidine + dorzolamide for three months when the initial intraocular pressure exceeded 14 mmHg. One physician prescribed carbidopa 25mg + levodopa 250mg T.I.D. for three months when Snellen visual acuity was worse than 20/200, either at onset or after progression. No other treatments were reported, including hyperbaric oxygen therapy or parenteral erythropoietin.

#### Recommendations for Lifestyle Modification

Thirty-one of 36 (86.1%) routinely recommended smoking cessation to smokers. Likewise, 29 (80.6%) advised men with NAION to avoid the use of erectile dysfunction medications, with 28 instructing this regardless of prior use. An additional 23 (63.8%) neuro-ophthalmologists referred patients to a low-vision clinic in certain indications, mostly (15/23) when poor vision existed in the fellow eye or when visual acuity was ≤ 20/200 in the afflicted eye (11/23).

## Discussion

Secondary prevention and management guidelines in NAION remain a challenge to implement. Known systemic and ophthalmological risk factors are associated with NAION, yet established guidelines on the choice of tests with which to establish the presence of these risk factors are lacking. Adding to this challenge is the fact that while neuro-ophthalmologists are well-versed as to the risk factors, many of the tests with which the presence of these risk factors are established, fall within the routine practice of general practitioners. This nationwide survey provides several insights into the practical approaches adopted by neuro-ophthalmologists and highlights the importance of developing a set of consensus guidelines on which specific tests should be ordered to evaluate diabetes, hypertension dyslipidemia, or OSA.

There is robust evidence supporting the association between NAION and cardiometabolic risk factors [22, 23]. Our findings reveal that there is a high level of agreement among neuro-ophthalmologists regarding *which* risk factors should be screened for after NAION. Approximately 80% of respondents recommended work-up for DM, HTN, dyslipidemia, and OSA. However, the specifics of how these conditions ought to be assessed varied between physicians. For example, an acute NAION patient who is not aware of harboring diabetes may be referred for; fasting glucose levels; a glucose tolerance test; serum HbA1c levels, or both. Similarly, screening for HTN may rely on periodic testing or referral for 24-h BP monitoring.

Nocturnal hypotension, defined as excessive dipping of systolic BP at nighttime, has been associated with NAION [24, 25]. Sixty-five% of neuro-ophthalmologists recommended a 24-h Holter BP monitoring. The topic of timing of anti-hypertensive medication to prevent nocturnal hypotension is challenging because while neuro-ophthalmologists often discourage taking antihypertensive medication at bedtime, the cardiovascular literature promotes bedtime antihypertensive dosing, emphasizing its potential to reduce cardiovascular risks [26, 27]. Labowsky et al. argued that prioritizing overall health (over vision) may justify bedtime antihypertensive medications even when nocturnal BP dipping may result [28]. Thus, routinely obtaining 24-h BP monitoring may not be universally productive.

Another issue of contention relates to the embolic workup of patients with NAION. While a substantial number of physicians in our study sent patients for embolic work-up, such as carotid Doppler sonography, it is crucial to consider that the presumed pathogenesis of NAION primarily involves hypoperfusion of the optic nerve head rather than thrombus or emboli. Embolic NAION case reports are extremely rare[29–31] and may lack evidence of causality. We contend that routine testing for internal carotid atheromatous plaque, cardiac valve thrombus, or arrhythmia is not recommended [1, 13] and may even deter primary care physicians from focusing on the most relevant risk factors and lifestyle modifications.

A specific challenge arises with screening for OSA. Several screening questionnaires for OSA with moderate sensitivity and specificity are available [32]. Fraser et al. recommended standardized and validated tools for OSA screening [33], highlighting that unstructured questions may decrease sensitivity and risk misdiagnosing this significant risk factor. Only a minority (11.1%) of respondents used the Berlin questionnaire following NAION, while 41.6% of neuro-ophthalmologists relied on unstructured OSA questions in their medical history. Given the high prevalence of OSA in NAION patients (55–89% [22]) and the limited sensitivity of existing screening questionnaires, it may be logical to consider unselectively referring all patients without a previous OSA diagnosis for polysomnography, as done by 41.6% of respondents in our study.

Screening for Ophthalmological risk factors also varied. While the association of optic disc drusen and NAION is well-documented [34], less than 40% routinely looked for optic disc drusen in NAION patients, nor was the type of imaging chosen to look for optic disc drusen uniform.

Over 91% of interviewees in our study recommended observation as the primary approach. Lifestyle and secondary prevention practices revealed a high level of inter-responder agreement. Unexpectedly, eighty percent of neuro-ophthalmologists recommended aspirin treatment, despite the paucity of evidence for its efficacy in reducing the risk for second-eye NAION [35]. Indeed, the largest study on this topic by Dr. Beck et al. retrospectively evaluated 431 patients experiencing their first NAION event. Among them, 153 who were prescribed aspirin and 278 who were not showed only a transient risk reduction for fellow-eye involvement within two years, with no long-term benefits observed [36]. Over 80% instructed their patients on the importance of lifestyle modifications (e.g. smoking cessation). Despite compelling evidence against the efficacy of high-dose steroids for NAION [6, 37, 38], 16% of respondents offered steroids in situations of second-eye involvement or progression. Our survey reveals that other therapeutic approaches, such as the administration of erythropoietin, have not gained widespread adoption in clinical practice.

In 2020, Lee et al. [16] conducted a similar survey on the management of NAION in South Korea. In their study, they evaluated the practice patterns of neuro-ophthalmologists in the diagnosis and management of NAION, traumatic optic neuropathy, and Leber's hereditary optic neuropathy. The authors sent a 15-question survey using Google Forms to all practicing neuro-ophthalmologists registered with the Korean Society of Neuro-ophthalmology. They had a higher number of responders (63) compared to our study (the population of South Korea is 5.5 times larger) but a lower response rate (78.8%). In their study, all neuro-ophthalmologists (100%) recommended routine neuro-imaging (an MRI) to patients suspected of having optic neuropathy compared to 22.2% in our study. Forty (63.5%) respondents recommended observation only, compared with more than 91% in our study. A much higher proportion of Korean neuro-ophthalmologists recommended corticosteroids in certain indications (38.1% gave IV steroids and an additional 20.6% gave P.O. steroids). Indications were variable and mainly included severe visual loss, fellow eye involvement, progressive visual loss, young age, old age, absence of vascular risk factors, and recurrent attack. Only 17.5% recommended aspirin (80.5% in our study). Eleven (17.5%) prescribed topical anti-glaucoma medications (5.5% in our study) and four (6.4%) treated patients with erythropoietin (none in our study). Comparing the two studies reveals significant disparities between countries. These findings underscore the ongoing debate surrounding the treatment of NAION.

This study has certain limitations. Like Lee et al. [16], the survey is confined to a single country and may not necessarily represent practices, which vary according to each country’s unique health-care system. Furthermore, unlike the Delphi method, a survey inherently lacks the structured iterative process necessary for achieving consensus among experts. However, it is crucial to clarify that our study was not designed to establish consensus guidelines for broader physician communities. Rather, we aimed to explore the prevailing approaches and the degree of controversy among neuro-ophthalmologists in managing patients with acute NAION. While a closed multiple-choice question format facilitated data compilation and uniformity, it may limit the depth of data acquisition. Results may also be influenced by the respondents' personal experiences and beliefs, which may diverge from evidence-based recommendations. Despite these limitations, this study had an excellent response rate of 97.3%, which was significantly higher than other surveys [15, 16] and the absence of missing responses was ensured through personal contact.

The conclusions drawn from this study are twofold. Firstly, the majority (~ 80%) of neuro-ophthalmologists find it significant to actively rule out DM, HTN, OSA, and dyslipidemia in NAION cases and avoid any treatment other than secondary prevention with aspirin. In forthcoming practice, neuro ophthalmologists may increasingly adhere to the continued use of aspirin following "the majority rule".

Secondly, contrary to the aforementioned agreement, there exists a considerable divergence in the preferred diagnostic approaches employed by clinicians. It appears that a prevailing sentiment persists that general practitioners may lack the requisite familiarity with NAION to conduct the appropriate workup. However, uniformity regarding the optimal diagnostic test for several risk factors remains lacking. Furthermore, our study highlights substantial disparities in clinical practices across different nations. These findings collectively emphasize the compelling need for the establishment of international guidelines for the evaluation and management of NAION. Adopting a uniform approach to diagnostic testing may help reduce the risk of second-eye involvement and overall patient disability, all while maintaining cost-effectiveness. From a legal perspective, adherence to standardized guidelines can mitigate the risk of malpractice claims by ensuring a consistent standard of care.

## Supplementary Information

Below is the link to the electronic supplementary material.

Supplementary file1 (DOCX 18 KB)

Supplementary file2 (DOCX 21 KB)
