# Supplementary material for: Evaluation and management of nonarteritic anterior ischemic optic neuropathy: a national survey
Source: Graefes Arch Clin Exp Ophthalmol. 2024 May 15;262(10):3323–30. doi: 10.1007/s00417-024-06512-y (PMC11458737; doi:10.1007/s00417-024-06512-y)
Supplement: Supplementary file 1 — Supplementary file1 (DOCX 18 KB) [file 417_2024_6512_MOESM1_ESM.docx]

**Evaluation and management of nonarteritic anterior ischemic optic neuropathy: a national survey**

Graefe's Archive for Clinical and Experimental Ophthalmology

Omer Y. Bialer^1,2^, Hadas Stiebel-Kalish^1,2^

1. Ophthalmology department, Rabin Medical Center, Petah-Tikva, Israel
2. School of Medicine, Tel-Aviv University, Tel-Aviv, Israel

Corresponding author e-mail: [Omer.bialer@gmail.com](mailto:Omer.bialer@gmail.com)

Supplemental Table 1 Characteristics of neuro-ophthalmologists in Israel

| Number of participants in the study 2019-2021 | 36^a^ |
| --- | --- |
| **Sex, n (%)** | |
| Women | 23 (63.9%) |
| Men | 13 (36.1%) |
| **Residency specialty, n (%)** | |
| Ophthalmology | 36 (100%) |
| **Place of fellowship in Neuro-ophthalmology, n (%)** | |
| United States of America | 24 (66.7%) |
| Israel | 10 (27.8%) |
| Australia | 1 (2.8%) |
| Switzerland | 1 (2.8%) |
| **Years of practicing Neuro-ophthalmology, years**^b^ | |
| Mean ± SD | 12.6 ± 9.1 |
| Range | 0 – 30 |
| < 5 years | 9 (25%) |
| 5 - 15 years | 13 (36.1%) |
| > 15 years | 14 (38.9%) |
| **Primary setting of practicing neuro-ophthalmology, n (%)** | |
| Hospital Ophthalmology department & clinic | 33 (91.7%) |
| Single practice (secondary care) clinic | 3 (8.3%) |
