# Supplementary material for: Evaluation and management of nonarteritic anterior ischemic optic neuropathy: a national survey
Source: Graefes Arch Clin Exp Ophthalmol. 2024 May 15;262(10):3323–30. doi: 10.1007/s00417-024-06512-y (PMC11458737; doi:10.1007/s00417-024-06512-y)
Supplement: Supplementary file 2 — Supplementary file2 (DOCX 21 KB) [file 417_2024_6512_MOESM2_ESM.docx]

**Evaluation and management of nonarteritic anterior ischemic optic neuropathy: a national survey**

Graefe's Archive for Clinical and Experimental Ophthalmology

Omer Y. Bialer^1,2^, Hadas Stiebel-Kalish^1,2^

1. Ophthalmology department, Rabin Medical Center, Petah-Tikva, Israel
2. School of Medicine, Tel-Aviv University, Tel-Aviv, Israel

Corresponding author e-mail: [Omer.bialer@gmail.com](mailto:Omer.bialer@gmail.com)

Supplemental Table 2 Questionnaire about the routine practices of

Neuro-Ophthalmologists in the management of patients with acute NAION

| Physician's name:  Date: | | | | |
| --- | --- | --- | --- | --- |
| **Do you routinely recommend or perform the following**  **investigations to all your patients with acute NAION:** | | | | |
|  |  | yes | no | Depends. If so, in what circumstances would you recommend this test? |
| 1 | Repeated self-measurement of BP at home? |  |  |  |
| 2 | 24-hour BP monitoring (BP Holter) |  |  |  |
| 3 | The Berlin questionnaire |  |  |  |
| 4 | Polysomnography |  |  |  |
| 5 | Fasting blood glucose |  |  |  |
| 6 | Serum Hemoglobin A1c level |  |  |  |
| 7 | Oral glucose tolerance test |  |  |  |
| 8 | Serum lipid profile |  |  |  |
| 9 | Ocular sonography to rule out optic nerve head drusen |  |  |  |
| 10 | EDI-OCT of the optic nerve head to rule out optic nerve head drusen |  |  |  |
| 11 | OCT of the optic nerve head to rule out vitreo-papillary traction |  |  |  |
| 12 | 24-hour electrocardiogram monitoring (ECG Holter monitor) |  |  |  |
| 13 | Carotid artery Doppler ultrasound |  |  |  |
| 14 | Echocardiogram |  |  |  |
| 15 | Serum homocystein test |  |  |  |
| 16 | Thrombophilia screen (such as protein C & S and Factor V Leiden) |  |  |  |
| 17 | Fluorescein angiography |  |  |  |
| 18 | CT of the head and orbits with contrast injection |  |  |  |
| 19 | MRI of the head and orbits with gadolinium |  |  |  |
| 20 | Other test: |  |  |  |
| **Do you routinely recommend the following treatments or**  **Lifestyle modifications to all your patients with acute NAION:** | | | | |
| 1 | Low dose aspirin |  |  |  |
| 2 | Smoking cessation |  |  |  |
| 3 | Avoiding erectile dysfunction medication for men |  |  |  |
| 4 | Oral or intravenous high-dose steroids |  |  |  |
| 5 | Referral to a low-vision clinic or service |  |  |  |
| 6 | Referral to welfare services for disability benefits |  |  |  |
| 7 | Other treatment: |  |  |  |
